# Closed-loop neuromuscular electrical stimulation using feedforward-feedback control and textile electrodes to regulate grasp force in quadriplegia

**DOI:** 10.1186/s42234-019-0034-y

**Published:** 2019-11-01

**Authors:** John Ciancibello, Kevin King, Milad Alizadeh Meghrazi, Subash Padmanaban, Todd Levy, Richard Ramdeo, Malgorzata Straka, Chad Bouton

**Affiliations:** 1 Feinstein Institute for Medical Research at Northwell Health, New York, USA; 2 Institute of Bioelectronic Medicine, Feinstein Institute for Medical Research, New York, USA; 3 Institute of Biomaterials and Biomedical Engineering, University of Toronto, Toronto, ON Canada; 4 Myant Corp, Toronto, ON Canada

## Abstract

**Background:**

Transcutaneous neuromuscular electrical stimulation is routinely used in physical rehabilitation and more recently in brain-computer interface applications for restoring movement in paralyzed limbs. Due to variable muscle responses to repeated or sustained stimulation, grasp force levels can change significantly over time. Here we develop and assess closed-loop methods to regulate individual finger forces to facilitate functional movement. We combined this approach with custom textile-based electrodes to form a light-weight, wearable device and evaluated in paralyzed study participants.

**Methods:**

A textile-based electrode sleeve was developed by the study team and Myant, Corp. (Toronto, ON, Canada) and evaluated in a study involving three able-body participants and two participants with quadriplegia. A feedforward-feedback control structure was designed and implemented to accurately maintain finger force levels in a quadriplegic study participant.

**Results:**

Individual finger flexion and extension movements, along with functional grasping, were evoked during neuromuscular electrical stimulation. Closed-loop control methods allowed accurate steady state performance (< 15% error) with a settling time of 0.67 s (SD = 0.42 s) for individual finger contact force in a participant with quadriplegia.

**Conclusions:**

Textile-based electrodes were identified to be a feasible alternative to conventional electrodes and facilitated individual finger movement and functional grasping. Furthermore, closed-loop methods demonstrated accurate control of individual finger flexion force. This approach may be a viable solution for enabling grasp force regulation in quadriplegia.

**Trial registration:**

NCT, NCT03385005. Registered Dec. 28, 2017

## Introduction

Paralysis affects nearly 5.4 million people in the US alone with the two leading causes being stroke and spinal cord injury (Armour et al. 2016). A survey of 681 persons with spinal cord injury revealed the top priority for individuals living with quadriplegia was regaining hand function (Anderson 2004). Currently, however, there is no FDA-cleared treatment or assistive device that can fully restore functional movement in individuals with complete hand paralysis.

Various invasive and non-invasive neuromodulation and neuromuscular electrical stimulation (NMES) devices have been employed to rehabilitate or evoke upper limb and hand movement (Sheffler and Chae 2007) (Huerta and Volpe 2009). Some devices, such as the Freehand System (which is no longer on the market), used shoulder movements coupled to switches that triggered several hand motions through electrical muscle stimulation via implanted electrodes (Cornwall and Hausman 2004). Cortical brain-computer interfaces (BCIs) have also been used to control NMES devices by recording and decoding motor activity in the brain to allow volitional control of an otherwise paralyzed hand (Bouton et al. 2016) (Ajiboye et al. 2017). Further refinements to this direct approach allowed graded control of joint torque and a reduced training burden as well (Sharma et al. 2016) (Friedenberg et al. 2017).

In invasive NMES devices, nerve cuffs or intramuscular electrodes are placed and connected via percutaneous leads or through wireless links. Hermetic packages are then used to house the electronics and a rechargeable battery, linked to the electrodes via wires. Inductive coupling is often used to power these devices or charge the on-board battery (Troyk et al. 2005). Other wireless powering/charging approaches being developed utilize high frequency radio waves or ultrasound to carry energy through the skin and tissue (Ho et al. 2014) (Seo et al. 2016).

To eliminate the need for surgery and to avoid the complications that can occur with implanted electrodes and electronic devices, non-invasive NMES devices have been developed and investigated (Bao et al. 2018; Bouton et al. 2016). The electrodes in these devices can be flexible or rigid and are often used with electrogels, hydrogels, or conductive creams to reduce skin impedance and thereby voltage required and discomfort during stimulation (Cooper et al. 2011). Arrays of over 100 small electrodes have been used on the forearm to transcutaneously stimulate hand muscles to achieve a wide variety of wrist and finger movements (Annetta et al. 2019). One limitation that remains for non-invasive NMES systems is that not all muscles can be reached effectively by the externally applied electric field resulting in a reduced or absent contraction. Another limitation of NMES devices is that they are often associated with rapid muscle fatigue and variable muscle contraction strength. To combat this, closed-loop control has been demonstrated to better regulate joint torques, such as in the ankle during foot movement (Frankel et al. 2016; Yoshida and Horch 1996). Closed-loop control of gross hand grasp force in the presence of disturbances has also been demonstrated (Lemay et al. 1993) (Crago et al. 1991). However, two remaining challenges are to accurately regulate individual finger contact forces during grasping to accommodate objects that are fragile or have complex shapes, and to develop a comfortable, wearable solution that can be used in a patient’s daily life. In this work we have developed an approach that accurately controls individual finger contact forces in a wearable, non-invasive form. This approach utilizes textile electrodes that can be worn for extended periods and may be incorporated in future neuromuscular stimulation device designs in assistive and rehabilitative applications for individuals living with paralysis or other motor impairments.

## Methods

Approval for this study was obtained from the Northwell Health Institutional Review Board (Great Neck, New York) and the study met institutional requirements for the conduct of human subject work. All participants completed an informed consent process and gave permission for obtaining photographs and videos during the study. Three able-body participants (two males and one female) and two male participants with quadriplegia participated. The able-body participants participated for 3–6 weeks each. Participant NMES04 was 55 years old at the time of the study, with a level of C5 on the American Spinal Injury Association (ASIA) impairment scale, A (complete), injury and participated in the study for a 2 month period. Participant NMES05 was 27 years old at the time of the study, with a C5 level, ASIA-A spinal cord injury and participated in the study for 5 months.

**Fig. 1:**
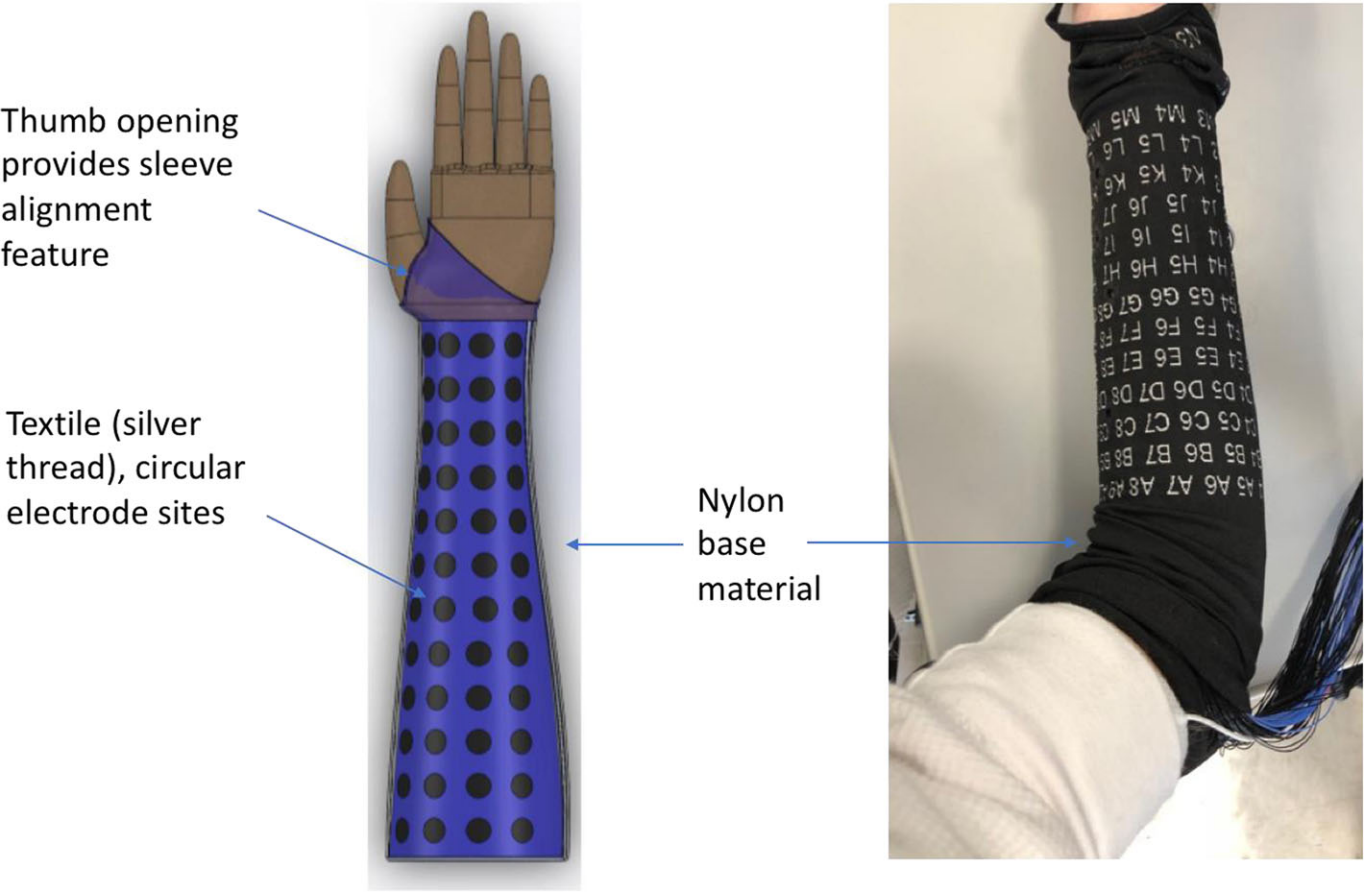
The sleeve is comprised of a nylon base material and silver thread forming circular electrode sites (128) that cover the forearm and facilitate stimulation of flexor and extensor muscles to produce a wide variety of wrist and hand movements

In this study, a textile-based electrode sleeve was developed by the study team and Myant Corp. (Toronto, ON, Canada) and evaluated in able-body and quadriplegic study participants. The sleeve is comprised of nylon materials commonly used in clothing and silver-thread to form circular conductive electrode sites with diameter of 12 mm for delivering electrical stimulation (see Fig. 1). The sleeve spans the forearm and has an opening for the thumb (see Fig. 1) and for the elbow (see Fig. 3a), which together allow proper and repeatable alignment of the electrode sites over muscles targets. The placement of the sleeve requires fewer steps and significantly fewer alignment points as compared to the electrode “cuff” described in Bouton et al. 2016, which has multiple fingers containing electrodes that must be positioned and wrapped around the forearm individually.

Electrical stimulation was applied to different electrode sites to evoke various movements. Symmetrical, bi-phasic (cathodic-leading) rectangular pulses at 50 Hz, with each phase being 500 μs in duration, were delivered manually by an operator to various electrode sites to evoke different types of wrist and individual finger movements. The SnR multichannel stimulator (Alpha Omega, Israel) was used for all experiments with a current amplitude ranging from 0 to 16 mA. With this maximum current, the SnR compliance voltage, and the size of the electrode, the power density was below 0.25 W/cm^2^ per FDA guidelines for transcutaneous muscle stimulators. Furthermore, during open-loop characterization, the operator ensured that the maximum current levels did not lead to discomfort or injury (and these maximum levels could not be exceeded during closed-loop mode operation).

Before each experiment, the participant’s arm was prepared with a conductive lotion from Theracream (Santa Barbara, CA), followed by a liberal application of Spectra 360 conductive gel from Parker (Fairfield, New Jersey). Current-regulated stimulation was applied in an open-loop manner (force was not regulated) to electrodes over various muscles (guided by known anatomy of the human forearm), while resulting movements were visually observed.

**Fig. 2:**
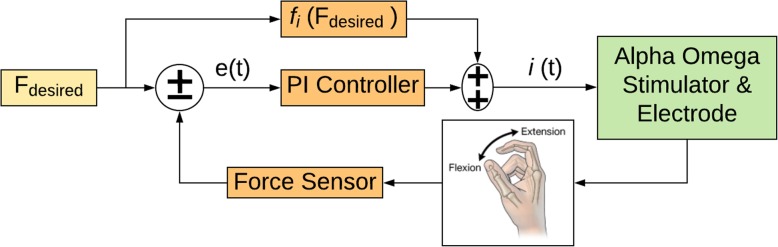
The feedforward-feedback control architecture included a feedforward block (f_i_) which uses the desired force (F_desired_) as an input to produce a computed current output. The output of the feedforward block is added to the feedback controller output and this sum is provided to the Alpha Omega Stimulator as the command input. The feedback controller is a proportional-integral (PI) type, providing current commands to the Alpha Omega stimulator

In the closed-loop experiments, the force sensor was calibrated and then positioned to measure middle finger flexion. Using custom software (Matlab), electrodes were identified for applying stimulation to evoke finger flexion movements. A stepwise increase in the desired force was input to the controller shown in Fig. 2, while the resulting force profile was recorded.

To allow regulated grasp force in participants with quadriplegia, a feedforward-feedback control architecture was implemented to provide closed-loop neuromuscular electrical stimulation as shown in Fig. 2 and described in Laplacian form as follows

$$ i(t)=f\left({F}_{desired}\right)+\left[{K}_p+\frac{K_i}{s}\right]\left[{F}_{desired}-{F}_{sensor}\right], $$

where f(F_desired_) is a linear function derived empirically by inverting and fitting force vs. current data when stimulation is applied to the forearm, K_p_ and K_i_ are the proportional and integral terms of the PI control algorithm, F_desired_ is the input command, and F_Sensor_ is the measured finger contact force.

A Model 31 force sensor from Honeywell (Morristown, New Jersey) was placed between the ring finger and palm during experiments, providing finger flexion force measurement and feedback to the controller. A desired force, F_desired_, was set by an operator and a step-shaped waveform was used in all experiments. The feedforward block consisted of a linear model derived empirically by stimulating the muscle at random current levels for a short time interval (approx. 2 s) and measuring the resulting force. For the feedback controller, a proportional-integral (PI) was used. The initial PI tuning parameters were determined through modeling (spring-mass-damper system) and simulation in Simulink by Mathworks, Inc. (Natick, Massachusetts). The tuning parameters were then further refined empirically during the session.

## Results and discussion

**Fig. 3:**
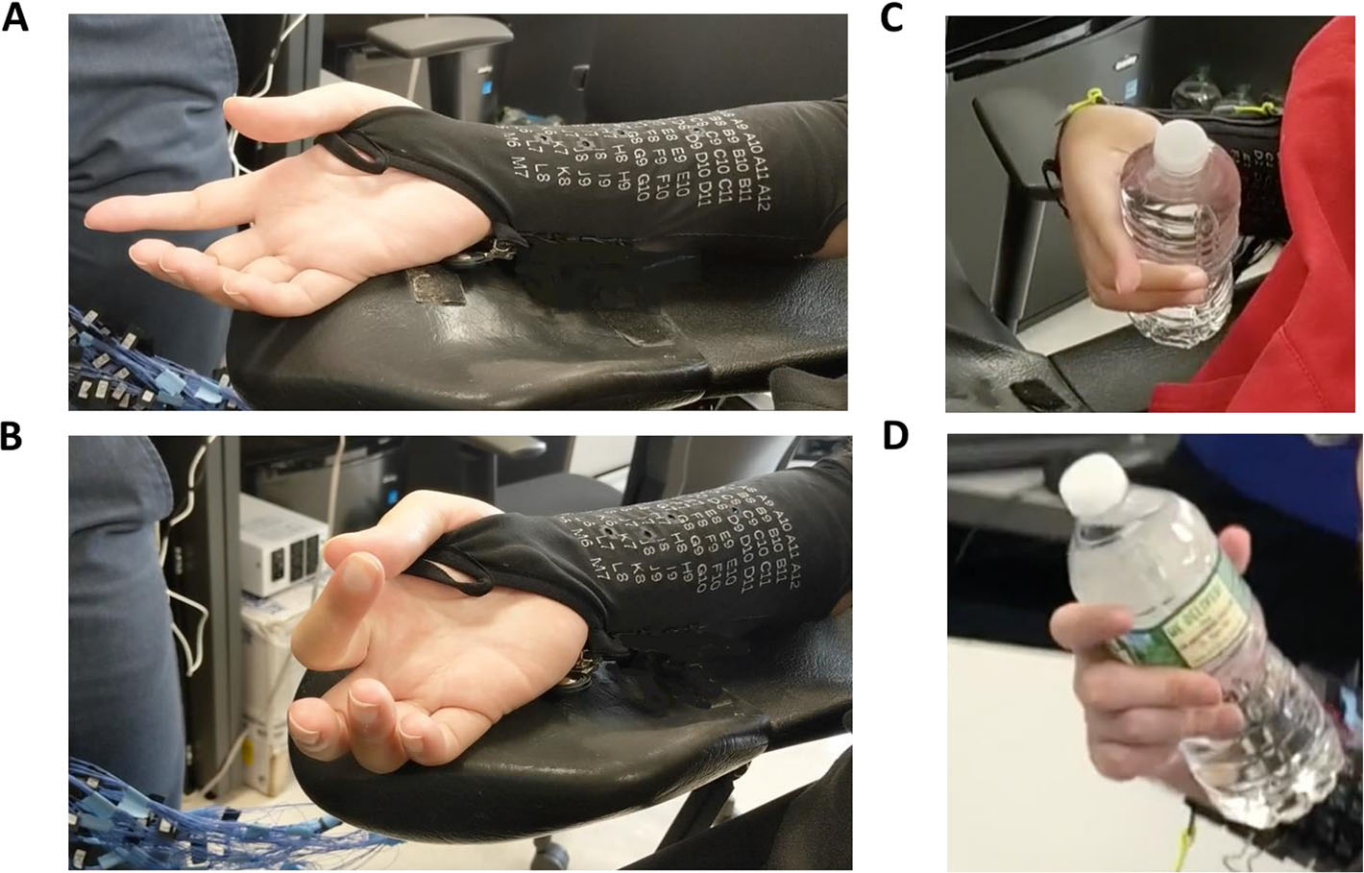
Textile-based electrodes in sleeve form. (**a**) Natural hand position when stimulation is off (**b**) Stimulation turned on, evoking index finger flexion and pinch-type movement (subject NMES05). (**c**) and (**d**) Stimulation evoked a cylindrical grasp with sufficient force to hold a full (750 mL) water bottle against gravity in two study participants with quadriplegia (NMES04 and NMES05 respectively)

In the first set of experiments, three able-body participants and two participants with quadriplegia received transcutaneous NMES stimulation in an open-loop fashion via textile electrodes to determine which hand movements were achievable. Table 1 summarizes finger flexion and extension movements, along with a cylindrical grasp, that were visually observed for each participant. Flexion movements in all digits were achieved in all three able-body participants, but not in the two participants with quadriplegia. Extension movement of the middle finger was not possible in the able-body participants, but was in both paralyzed participants. Mixed results were obtained for the ring and pinky fingers. Transcutaneous stimulation via the textile electrode sleeve developed in the study was also effective in evoking a cylindrical grasp in all participants. This type of movement was further assessed with real-world objects including a full (750 mL) water bottle, which as shown in Fig. 3 along with an index flexion movement as well. Finally, it was observed that varying contact pressure between the textile electrodes and the skin could impact muscle contraction intensity. Devices, such as mechanical clips along the seam of the sleeve, were used to increase its conformity to the skin. This suggests the fit of the sleeve is an important performance factor.

**Table 1:** Hand movements visually observed in able-body (A) and participants with quadriplegia (Q)

Hand Movements											
Participant	Thumb		Index		Middle		Ring		Pinky		Cyl. Grasp
	Flex.	Ext.	Flex.	Ext.	Flex.	Ext.	Flex.	Ext.	Flex.	Ext.	
NMES01 (A)	•	•	•	•	•		•	•	•	•	•
NMES02 (A)	•	•	•	•	•		•		•		•
NMES03 (A)	•	•	•	•	•		•		•	•	•
NMES04 (Q)			•	•	•	•	•	•		•	•
NMES05 (Q)	•	•	•	•	•	•	•				•

**Fig. 4:**
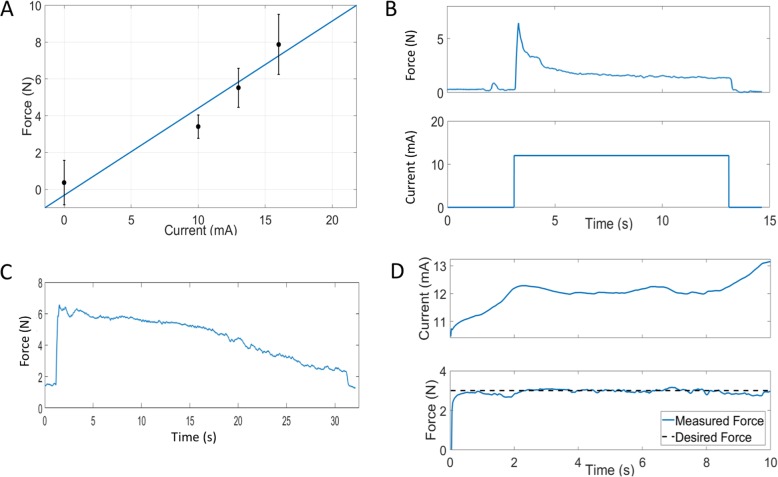
Open and closed-loop responses during neuromuscular stimulation. **a** Force produced for various stimulation amplitudes and linear fit (blue). **b** and **c** Example open-loop responses to neuromuscular electrical stimulation. **d** Representative closed-loop response to a desired force step input; between 8 and 10s the controller automatically increases the current delivered to the target muscle to compensate for muscle fatigue

To assess the feasibility of closed-loop neuromuscular stimulation using a feedforward-feedback control structure, a series of tests were conducted in a participant with quadriplegia (NMES05) having a C5 level ASIA-A spinal cord injury. First, the muscle response was characterized by measuring the force exerted by the target finger for various stimulation current levels (leading phase amplitude). This was modeled as a linear function of the stimulation current as shown in Fig. 4a. Note, the non-zero force values measured when the current is zero were caused by slight physical contact between the finger and the force transducer which was present prior to stimulation began. Next, fatigue effects were examined by recording force during extended stimulation periods. Representative traces of force patterns during constant (open-loop) stimulation are shown in Fig. 4b and c. These demonstrate that open-loop/feedforward methods yield large overshoots and/or significant drift over time, both more than 50%. To address these fatigue-induced issues, the closed-loop approach previously described was implemented and tested. Figure 4d shows the rapid (approx. 0.1 s) and stable force response when employing this method. For this particular test, it was observed that the controller automatically increased the current in response to a decrease in force toward the end of the 10s test.

**Fig. 5:**
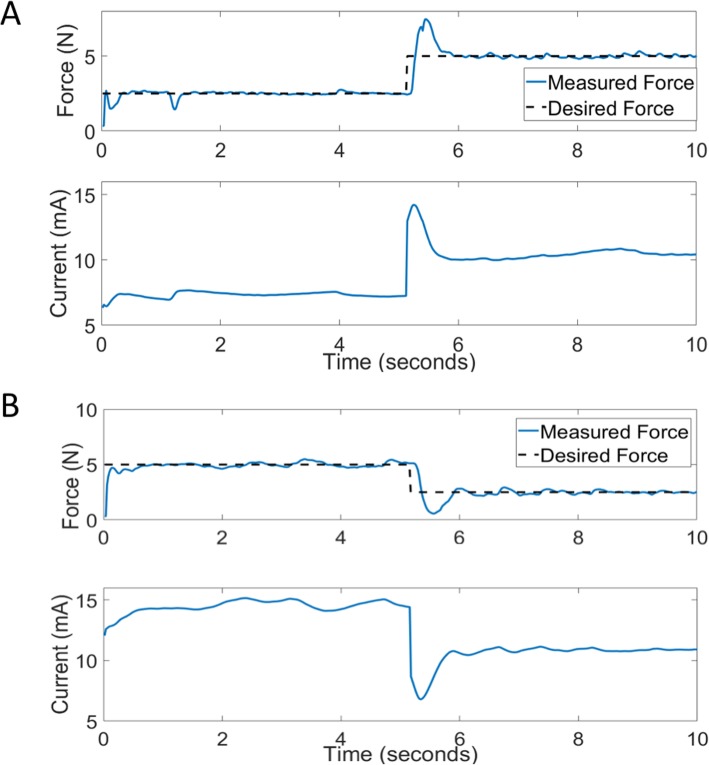
Closed-loop response to increasing and decreasing target (desired) forces. **a** The target force is increased from 2.5 to 5 N causing a rapid response that overshoots the final desired value, but settles within +/− 10% of the final value in less than 1 s. **b** The target force is decreased from 5 to 2.5 N associated with a rapid response, settling within 1 s

To assess the ability of the control algorithm to track changing desired force levels, a variety of step inputs were used as the desired force profiles. As shown in Fig. 5a, the response to a positive step input was rapid and stable. Furthermore, the response to a negative-going step input is shown in Fig. 5b.

During 14 sessions, over a 5 month period, refinement of the control algorithm’s tuning parameters was performed and repeatable and stable closed-loop performance was achieved. Towards the beginning of this period, the mean settling time (time required to reach and stay within +/− 15% of the desired force) was 0.67 s (SD = 0.42 s). By the end of this 5 month period, performance had been improved, achieving a settling time of 0.135 s (SD = 0.054 s). These results demonstrate that accurate regulation of force is achievable with the control architecture described.

Our results show that a feedforward-feedback closed-loop control approach can be used in combination with light-weight textile electrodes to provide precise grasp force regulation in quadriplegia and suggests this may be a viable approach for assistive or rehabilitative applications and portable use outside the laboratory. In three able-body participants and two participants with quadriplegia, our wearable device evoked flexion and extension finger movements, along with a cylindrical grasp, in all participants. Furthermore, the hand grasping produced in the participants with quadriplegia could support a full water bottle, thereby demonstrating potentially utility in daily living.

In our study, closed-loop methods for individual finger force regulation were evaluated. The force reached a steady-state value that was within 15% of the desired force with a mean settling time that ranged from 0.135 s (SD = 0.054 s) to 0.67 s (SD = 0.42 s). Previous research demonstrated a settling time of 4.5 s for closed-loop joint position control of the hind-limb ankle joint in a feline (Frankel et al. 2016). Also, response rise times of less than 2 s were reported in studies involving hand grasp force regulation (Crago et al. 1991). In feedback-only control structures, the controller gain parameters and therefore the aggressiveness of the controller can be limited, whereas in our current work, using feedforward compensation allowed higher gain values and settling times of less than 1 s were achieved.

One of the limitations in our study is that only one finger was controlled and calibration by a trained operator is required. Although single finger force regulation demonstrates the ability to isolate and control an individual finger with the control algorithm developed and small textile electrodes, controlling contact forces for multiple fingers during grasping of complex objects will require a more sophisticated control algorithm. Therefore, the development of such algorithms and automatic calibration are planned for future studies where their performance will be evaluated. Furthermore, it was observed that the contact pressure between the textile electrodes and the skin can affect the resulting forces that are generated at the fingers and cause variability. In future studies, changes are planned for the sleeve design to address this limitation. Finally, the force setpoint (input to the controller) must ultimately be derived from the user’s intentions as they initiate and complete grasping operations. In the future, we plan to integrate the methods and technology presented in this paper with a brain-computer interface which will decode (decipher) desired grasping forces. These decoded intentions would then be translated into setpoint commands that are fed into the controller to facilitate accurately regulated hand movements.

## Conclusions

Textile-based electrodes were identified to be a feasible alternative to conventional electrodes and facilitated individual finger movement and functional grasping. Furthermore, closed-loop methods demonstrated accurate control of individual finger flexion force which may be a viable solution for enabling grasp force regulation in quadriplegia. These findings warrant future investigation of using multiple force sensors on additional fingers and more sophisticated control architectures to facilitate manipulation of a wide variety of real-world objects. These new closed-loop methods, automatic calibration, and a wearable textile-based electrode sleeve, may allow users living with quadriplegia to perform functional tasks with their hands outside the laboratory in the future. This would have a positive impact on the quality of daily living and increase the level of independence achieved.

## Data Availability

Please contact the corresponding author for data requests.

## Abbreviations

ASIA: American Spinal Injury Association
NMES: Neuromuscular electrical stimulation
SD: Standard deviation
